# Pigmented Contact Dermatitis to Cosmetics: A Prospective Observational Study From a Tertiary Care Hospital

**DOI:** 10.7759/cureus.113544

**Published:** 2026-07-28

**Authors:** Sania Khan, Ram Chander, Amrit Pant, Vijay K Garg, Somya Sharma, Gulshant Panesar

**Affiliations:** 1 Dermatology, Santosh University, Ghaziabad, IND; 2 Dermatology, Santosh Medical College, Ghaziabad, IND; 3 Dermatology, Vinayak Skin Clinic, Delhi, IND

**Keywords:** cosmetic allergy, dermoscopy, facial melanosis, indian standard battery, paraphenylenediamine, patch testing, pigmented contact dermatitis

## Abstract

Background

Pigmented contact dermatitis (PCD) is a pigmentary variant of contact dermatitis characterized by gradually developing facial hyperpigmentation with minimal or absent overt dermatitis. Owing to its close resemblance to other causes of facial melanosis, PCD is frequently underdiagnosed. Patch testing plays a crucial role in identifying causative allergens and guiding avoidance-based management.

Objectives

To evaluate the clinical profile, dermoscopic features, and patch test allergen spectrum in patients with PCD attending a tertiary care hospital.

Methods

This prospective observational study was conducted over a period of 1.5 years in the Department of Dermatology, Santosh Medical College and Hospital, Ghaziabad. A total of 55 patients clinically diagnosed with PCD and fulfilling inclusion criteria were enrolled after informed consent. Detailed demographic and clinical data were recorded using a structured proforma. Dermatoscopic examination was performed using a DermLite DL5 dermoscope (DermLite LLC, Aliso Viejo, CA). Patch testing was carried out using the Indian Standard Battery and Indian Cosmetic Series, with readings taken at 48 hours, 96 hours, and on day 7, interpreted according to ICDRG criteria. Statistical analysis was performed using IBM SPSS version 27.0.

Results

The majority of patients were women (n=41; 74.5%), with the commonest age group being 31-40 years (n=23; 41.8%). Brownish-black pigmentation was the predominant clinical presentation. Dermatoscopy most frequently revealed brownish-gray background pigmentation, brown and gray globules, and follicular plugs with perifollicular white halos. Patch test positivity to at least one allergen was observed in 43 (78.2%) patients. Paraphenylenediamine (n=16; 27.1%) was the most common allergen, followed by cetrimide (n=9; 15.3%) and lavender absolute (n=7; 11.9%). Hair dye was the most frequent exposure source associated with allergen positivity.

Conclusion:

PCD predominantly affects young to middle-aged females and is strongly associated with cosmetic and hair dye exposure. Patch testing is an essential diagnostic tool for identifying relevant allergens and enabling effective prevention through targeted avoidance.

## Introduction

Pigmented contact dermatitis (PCD) is an acquired hyperpigmentation disorder in which repeated exposure to external contact allergens--most often cosmetics, fragrances, hair dyes, and traditional topical products--leads to gradual brown to grey-brown pigmentation with little to no overt eczematous dermatitis. It is seen more commonly in darker skin phototypes and shows a female predominance, likely reflecting cumulative exposure to personal-care products and/or cultural practices. Clinically, PCD presents as diffuse or reticulated pigmentation, classically involving the face and sometimes extending to the neck and upper chest, with mild burning or pruritus but minimal erythema or scaling. In contemporary dermatology, PCD is discussed within the spectrum of acquired dermal macular hyperpigmentation (ADMH) and overlaps with Riehl melanosis; however, PCD is distinguished by the demonstration of an external allergen, typically through patch testing [1].

The term “pigmented contact dermatitis” was introduced by Osmundsen after describing an outbreak of melanosis and highlighting the role of repeated, low-grade exposure to everyday products [2]. Also referred to as pigmented cosmetic dermatitis or Riehl’s melanosis, this condition represents a non-eczematous pattern of contact dermatitis where pigmentation is the dominant clinical feature [3]. Common triggers include fragrances, hair dyes, creams, and sunscreens, as well as culturally used decorative agents such as kumkum and sindoor, where chronic exposure can act as a key driver of persistent facial hyperpigmentation [4-7]. In Indian patients, pigmentary changes are often more conspicuous and may cause considerable cosmetic concern and psychological impact [8].

Pathogenetically, PCD is primarily a delayed-type (Type IV) hypersensitivity reaction. Repeated exposure to weak allergens produces subtle but persistent inflammation, basal cell damage, and melanin incontinence, resulting in pigment deposition in the superficial dermis despite minimal clinically visible dermatitis [9-11]. PCD closely mimics other causes of facial hyperpigmentation such as lichen planus pigmentosus, erythema dyschromicum perstans, and melasma; underdiagnosis and delayed treatment are common [10]. Patch testing is therefore considered the diagnostic gold standard, as it helps to identify the causative allergen and guides targeted avoidance--an essential step because residual pigmentation may persist for months even after allergen withdrawal [12]. Dermoscopy in PCD reveals a characteristic brownish-grey background with fine grey-brown dots and globules arranged in reticular, arcuate, or honeycomb patterns, accompanied by telangiectasias and follicular plugs [1].

With increasing long-term use of personal-care products, PCD is being encountered more frequently in tertiary-care practice [13]. The allergen spectrum varies by region and product-use patterns; in India, commonly implicated sensitizers include paraphenylenediamine, fragrances, and preservatives, alongside culturally relevant exposures such as kumkum [10,14-18]. Given the limited number of large, systematic Indian studies focused specifically on PCD, documenting clinico-demographic patterns and locally relevant allergens are important for improving recognition, strengthening diagnostic protocols, and enabling effective prevention through patient education and allergen avoidance [7,8,19,20].

The present tertiary-care study aimed to characterize the clinical profile and identify common cosmetic allergens causing PCD using patch test correlations, thereby strengthening recognition and management in routine practice.

## Materials and methods

Study design and setting

This was an institution-based prospective observational study conducted in the dermatology OPD of Santosh Medical College, Ghaziabad, Uttar Pradesh, over a study period of 1.5 years from June 2024 to December 2025.

Study population

Consecutive patients attending the dermatology outpatient clinic and clinically diagnosed with PCD were screened. Eligible participants fulfilling the inclusion and exclusion criteria were enrolled after obtaining written informed consent.

Sample size calculation

Sample size was calculated using Cochran’s formula for estimating a proportion, based on a previous study in which patch test positivity was noted in 3.4% of total patients [7].

$n =\frac{Z^{2}pq}{d^{2}}$

where n is the required minimum sample size, Z = 1.96 at the 95% confidence interval, p = 0.034, q = 1 − 0.034 = 0.966, and d is the absolute error (5% = 0.05). Thus, a minimum sample size of 50 participants was required.

Inclusion criteria

Patients over 18 years of age presenting with facial hyperpigmentation with minimal or absent inflammatory component and a history suggestive of exposure to cosmetics as a probable allergen source, clinically diagnosed as PCD, and willing to provide informed written consent were included in the study.

Exclusion criteria

Patients with congenital hypermelanosis, melasma, systemic illnesses, or a doubtful clinical diagnosis were excluded from the study. Individuals unwilling to provide informed consent were also excluded. In addition, patients receiving oral corticosteroids or other immunosuppressive medications, pregnant or lactating females, and immunocompromised individuals, including those with HIV infection, were excluded.

Data collection and clinical assessment

A structured proforma was used to record demographic and clinical details, including age, sex, religion, occupation, and residence. A detailed history of onset, duration, progression, and aggravating factors was obtained with specific documentation of exposure to cosmetics and personal-care products (fragrances, hair dyes, oils, soaps, textiles, and toiletries). Past medical history and other relevant findings were also recorded. Photographic assessment and dermatoscopy were performed using a handheld DermLite DL5 dermatoscope (DermLite LLC, Aliso Viejo, CA) and Apple iPhone 15 Pro (Apple Inc., Cupertino, CA).

Patch Testing Procedure

Patch testing was performed using standardised allergens from the Indian Standard Series and Indian Cosmetic Series (Systopic Laboratories Pvt. Ltd., New Delhi, India) [21, 22]. The allergens were tested on the upper back of the patient using the Finn Chambers on Scanpor.

Readings were recorded at 48 hours, 96 hours, and on Day 7 and graded according to the International Contact Dermatitis Research Group (ICDRG) descriptive grading scale (1970) [23].

Reactions were graded as "−" negative (no reaction), "? or IR" (doubtful/irritant; macular or faint erythema only, lacking the hallmarks of a true allergic response), "+" (weak positive; nonvesicular, characterized by slight redness, infiltration (palpable thickness), and possibly small papules), "++" (strong positive; vesicular, with redness, swelling (edema), and clearly visible blisters or vesicles), or "+++" (extreme positive; bullous or ulcerative, with a severe reaction, large spreading blisters, or tissue destruction extending beyond the original application site). Findings were documented in the proforma and photographs were taken at each reading.

Statistical analysis

Data were entered and analysed using IBM SPSS Statistics v. 29.0 (IBM Corp., Armonk, NY). Descriptive statistics were used to summarise variables (frequency tables, graphs). Appropriate parametric and non-parametric tests (Pearson chi-square test) were applied based on data distribution and variable type. A p-value <0.05 was considered statistically significant.

## Results

**Figure 1:**
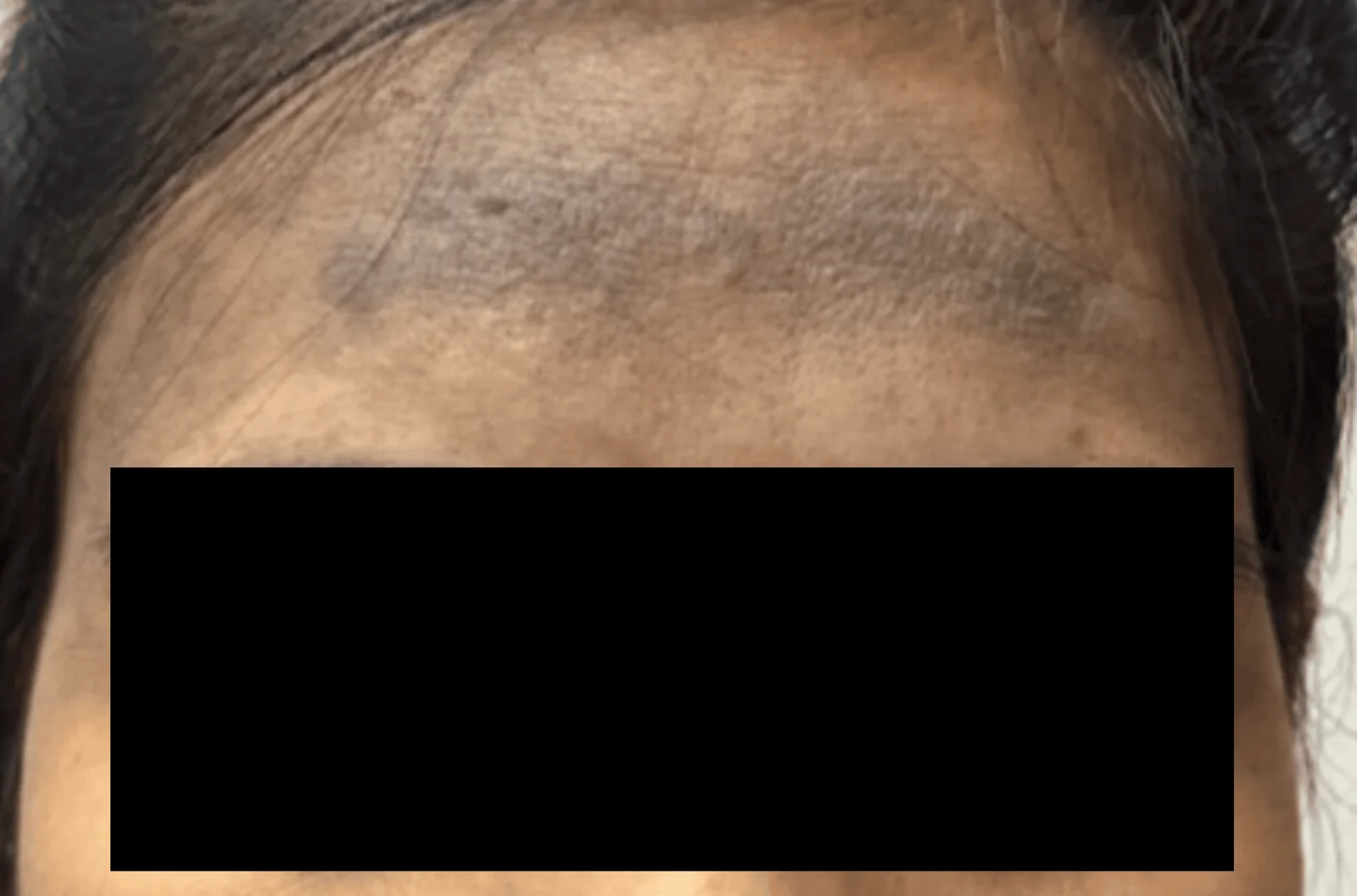
Bluish-black hyperpigmentation on forehead due to topical analgesic cream Bluish-black colour of pigmentation on the forehead of a patient who had a history of chronic use of topical analgesic cream.

**Figure 2:**
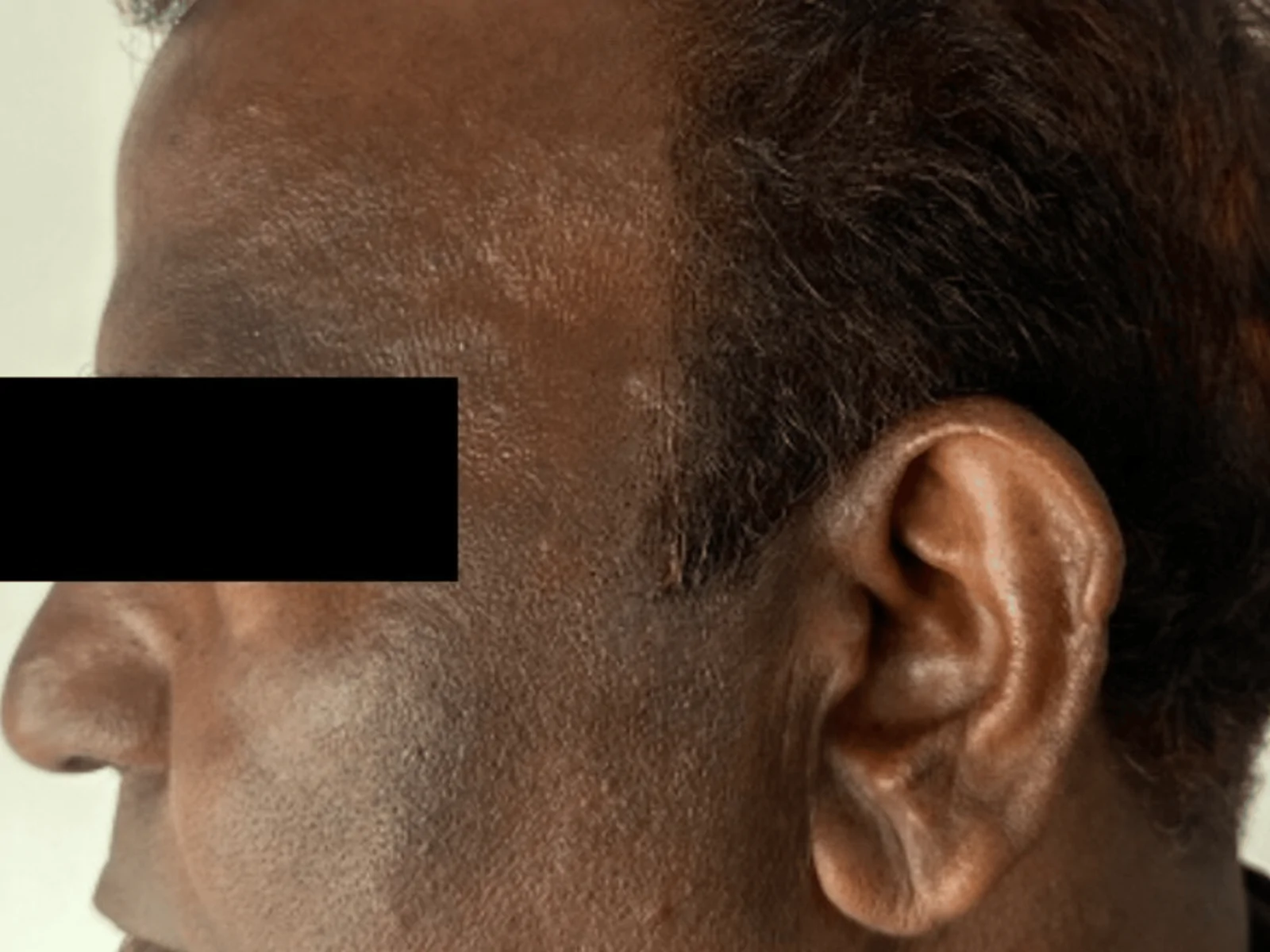
Diffuse brownish-black hyperpigmentation on forehead, temples, pinna and side of the face due to hair dye. Brownish-black hyperpigmentation is the most common presentation of colour in pigmented contact dermatitis.

In this cohort, PCD was most frequently observed in those aged 31-40 years (n=23; 41.8%), indicating clustering in this age group. Females constituted nearly three-fourths of patients (n=41; 74.5%), consistent with greater cosmetic and personal-care product exposure. Most participants were married (n=51; 92.7%), which may reflect both the age structure of the sample and longer cumulative exposure to household and cosmetic allergens (Table 1). The commonest pigmentation was brownish-black (n=29; 52.7%), followed by dark brown (n=20; 36.3%), while bluish-black pigmentation was relatively uncommon (n=6; 10.9%). Violaceous/slate-grey lesions were absent in our study population (Table 2). Figures 1-2 depict different colour morphologies of PCD lesions.

**Table 1:** Socio-demographic profile of patients with pigmented contact dermatitis (total patients, n=55).

Variable	Category	Frequency (n)	Percent (%)
Age (years)	21-30	7	12.7
	31-40	23	41.8
	41-50	15	27.3
	51-60	10	18.2
Sex	Female	41	74.5
	Male	14	25.5
Marital status	Married	51	92.7
	Unmarried	3	5.5
	Divorced/widow	1	1.8

**Table 2:** Color pattern of lesions in patients with pigmented contact dermatitis (total patients, n=55). Brownish black is the dominant category, over half the sample. Dark brown is second most common. Bluish black is rare, and violaceous/slate grey doesn't appear at all.

Color	Frequency (n)	%
Violaceous / Slate Grey	0	0
Bluish-black	6	10.90
Brownish-black	29	52.70
Dark brown	20	36.40

Dermatoscopy showed brownish-grey background pigmentation in 98.2% of cases, brown and grey globules in 92.7%, and follicular plugs with perifollicular white halos in 90.9%. Linear vessels were seen in 41.8%, superimposition of brown globules in 32.7%, and mild surface scaling in 25.5% of patients (Table 3).

**Table 3:** Dermatoscopic findings in patients with pigmented contact dermatitis (total patients, n=55).

Dermatoscopic Feature	Finding (n)	%
Brownish-grey background pigmentation	54	98.2
Brown and grey globules / pigment pattern	51	92.7
Linear vessels	23	41.8
Mild surface scaling	14	25.5
Follicular plugs with perifollicular white halos	50	90.9
Superimposition of brown globules	18	32.7

Of the 55 patients who underwent patch testing, 43 (78.2%) were positive to at least one allergen, while 12 (21.8%) were negative to all tested allergens. In the present study, patch testing was performed in 55 patients using the Indian Standard Battery (ISB) in combination with the Indian Cosmetic Series (ICS). A total of 2,850 patches were applied. A total of 59 positive patch reactions were observed, resulting in a patch-based positivity rate of 2.07% (Table 4).

**Table 4:** Patch test positivity status Out of 55 patients, there were 59 positive patch reactions. The Indian Standard Battery and Cosmetic Series have 20 and 32 allergens, respectively, so a total of 55 allergens were tested in all individuals, out of which 43 patients showed a positive reaction to at least one allergen.

Variable	Category / Parameter	Value
Overall patch test status	At least one allergen positive, n (%)	43 (78.2)
	Completely negative to all allergens, n (%)	12 (21.8)
	Total, n (%)	55 (100.0)
Patch testing details	Patch test series used	Indian Standard Battery (ISB) + Indian Cosmetic Series (ICS)
	Total patches applied	2,850
	Total positive patch reactions	59
	Patch-based positivity (%)	2.07%

Among the 59 positive patch test reactions recorded in the study, paraphenylenediamine (PPD) was the most frequently implicated allergen, accounting for 27.1% of all positive reactions, followed by cetrimide (15.2%) and lavender absolute (11.8%). The remaining allergens were observed at lower frequencies, each contributing less than 10% of total reactions (Table 5).

**Table 5:** Distribution of allergen among positive patch test reactions (total patients=55, total positive patch test reactions=59)

Allergen	Positive reactions (n)	%
Paraphenylenediamine (PPD)	16	27.10
Cetrimide	9	15.20
Lavender absolute	7	11.80
Rose oil	5	8.40
Geranium oil	4	6.70
Neomycin	2	3.30
Ethylenediamine	2	3.30
Sorbitan sesquioleate	2	3.30
Vanillin	2	3.30
Parthenium	1	1.60
Chlorocresol	1	1.60
Fragrance mix	1	1.60
Musk mix	1	1.60
Benzyl salicylate	1	1.60
Butylated hydroxyanisole (BHA)	1	1.60
Potassium bichromate	1	1.60
Cetyl alcohol	1	1.60
Thiuram mix	1	1.60
Rubber mix	1	1.60

The analysis of allergen-specific clinical relevance did not reveal a statistically significant association (Pearson χ²=39.46, df=40, p=0.494). Most allergens were classified as weakly positive, suggesting likely but minimal clinical significance. Strongly positive reactions were not very prevalent and were mostly seen with PPD and rose oil, which is in line with what is known about their ability to cause allergies. A minor fraction of allergens was classified as no reaction, indicating either prior sensitisation lacking current significance or irritating responses (Table 6).

**Table 6:** Agent-wise clinical relevance pattern

Allergen (Agent)	No reaction	Strongly positive	Weak positive	Total
Paraphenylenediamine (PPD)	0 (0.0)	3 (18.8)	13 (81.3)	16
Cetrimide	1 (11.1)	0 (0.0)	8 (88.9)	9
Lavender absolute	2 (28.6)	0 (0.0)	5 (71.4)	7
Rose oil	0 (0.0)	1 (20.0)	4 (80.0)	5
Geranium oil	0 (0.0)	0 (0.0)	4 (100.0)	4
Neomycin	0 (0.0)	0 (0.0)	2 (100.0)	2
Ethylenediamine	0 (0.0)	0 (0.0)	2 (100.0)	2
Sorbitan sesquioleate	1 (50.0)	0 (0.0)	1 (50.0)	2
Vanillin	0 (0.0)	0 (0.0)	2 (100.0)	2
Chlorocresol	0 (0.0)	0 (0.0)	1 (100.0)	1
Parthenium	0 (0.0)	0 (0.0)	1 (100.0)	1
Fragrance mix	0 (0.0)	0 (0.0)	1 (100.0)	1
Musk mix	0 (0.0)	0 (0.0)	1 (100.0)	1
Thiuram mix	0 (0.0)	0 (0.0)	1 (100.0)	1
Rubber mix	0 (0.0)	0 (0.0)	1 (100.0)	1
Benzyl salicylate	0 (0.0)	0 (0.0)	1 (100.0)	1
Butylated hydroxyanisole (BHA)	0 (0.0)	0 (0.0)	1 (100.0)	1
Potassium bichromate	0 (0.0)	0 (0.0)	1 (100.0)	1
Cetyl alcohol	0 (0.0)	0 (0.0)	1 (100.0)	1

**Figure 3:**
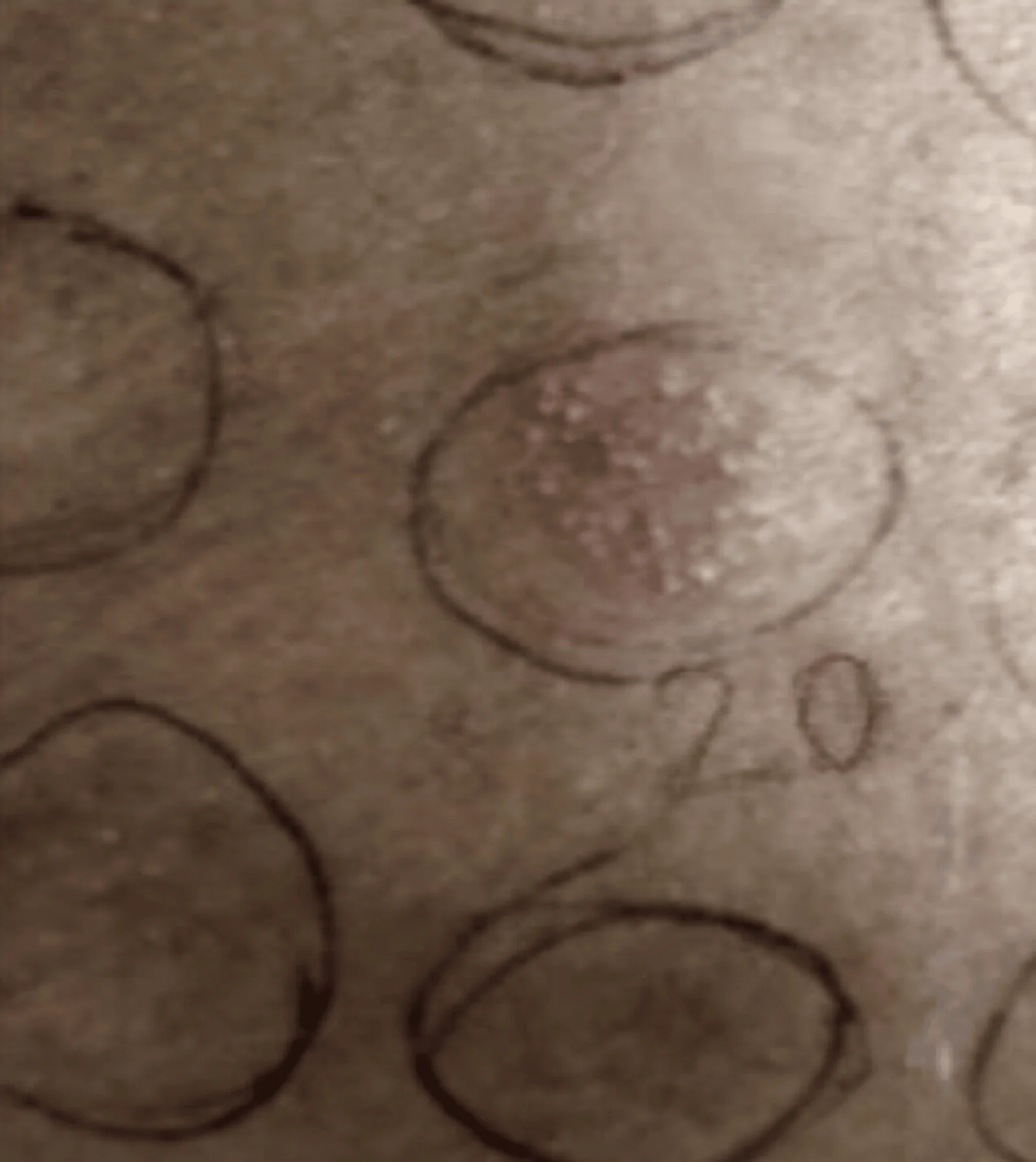
Indian Cosmetic Series patch test showing grade 2 (ICDRG) positivity for rose oil (allergen 20)

**Figure 4:**
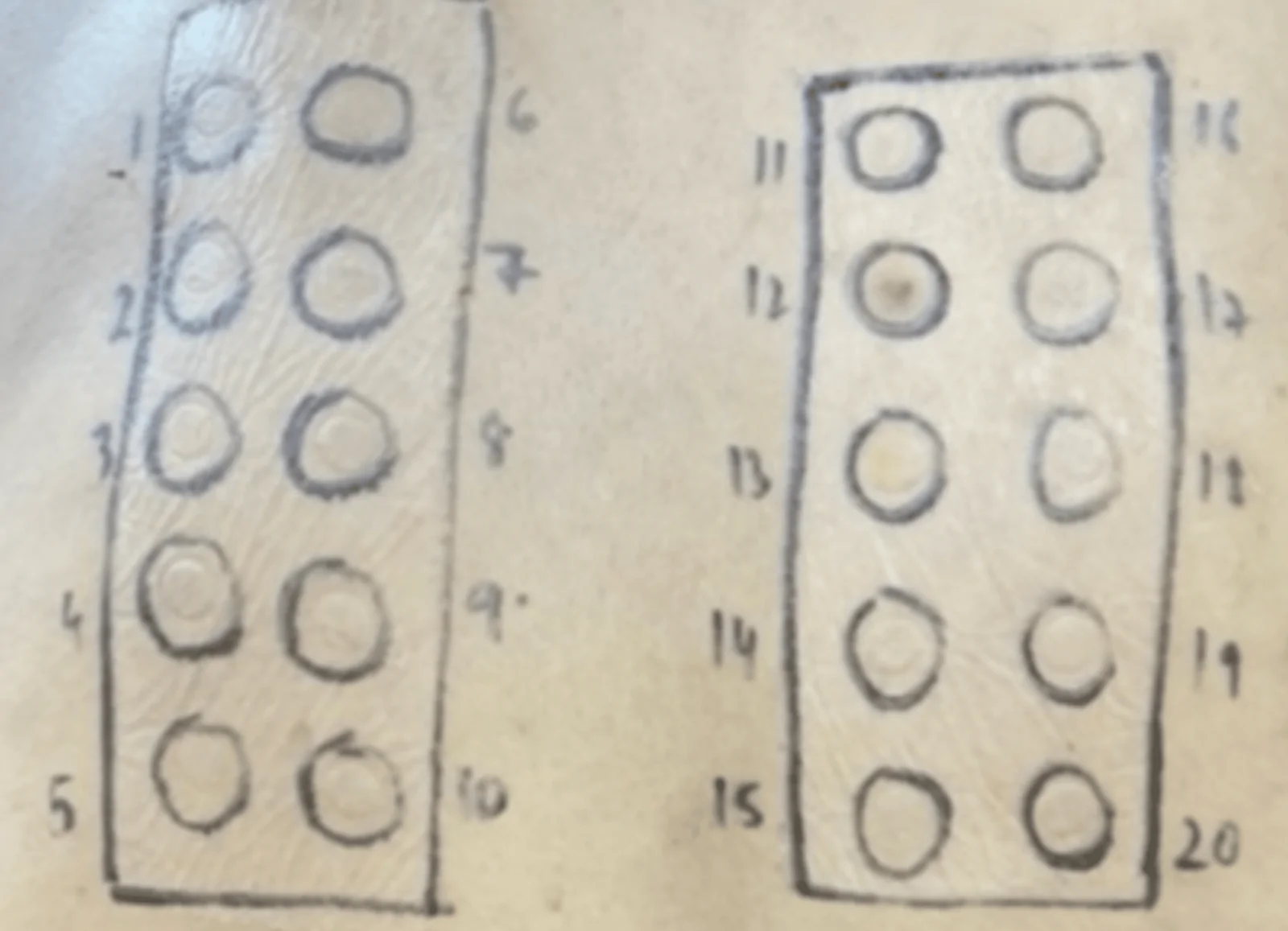
Indian Standard Battery patch test at 48 hours showing grade 1 (ICDRG) positivity for paraphenylenediamine (allergen 12)

Figure 3 depicts a strongly positive (++) reaction noted for rose oil, and Figure 4 depicts a weakly positive reaction (+), with PPD, suggesting a hyperpigmented macule is considered a positive result and may not always present with an obvious papule, vesicle, or irritant reaction.

Among the 59 patch-test-positive reactions, hair dye exposure was the most frequent source of allergen positivity, identified in 33 cases (55.9%), followed by fairness creams in 24 cases (40.7%), cosmetic products in 22 cases (37.3%), and mehendi (henna) in 21 cases (35.6%). Skincare products accounted for a small proportion of reactions (8 cases; 13.6%), while hair products were hardly implicated (1 case; 1.7%).

Paraphenylenediamine was the most common allergen overall (27.1%), with strong associations with hair dye, mehendi, fairness creams, and cosmetic products. Other frequently implicated allergens included cetrimide (15.3%), lavender absolute (11.9%), rose oil (8.5%), and geranium oil (6.8%) (Table 7).

**Table 7:** Distribution of patch test-positive allergens across exposure sources Total number of patients=55; total number of patients with positivity to at least one allergen=43; N=59 is the total number of positive patch reactions, signifying that some individuals showed positivity to more than one allergen. Some patients did report multiple exposure sources, like application of hair dye as well as perfumes in the same individual. Hair dye consists of all possible dyes that have been used to colour the hair; various oils in the Indian market are marketed as hair colouring agents. Hair products include shampoos, conditioners, styling products, oils, hair serum, gels, and sprays.

Allergen (Agent)	Hair dye	Fairness creams	Cosmetic	Mehendi	Skincare	Hair products
Paraphenylenediamine (n = 16)	9 (56.3)	8 (50.0)	8 (50.0)	9 (56.3)	0 (0.0)	0 (0.0)
Cetrimide (n = 9)	6 (66.7)	3 (33.3)	2 (22.2)	3 (33.3)	1 (11.1)	0 (0.0)
Lavender absolute (n = 7)	4 (57.1)	5 (71.4)	0 (0.0)	1 (14.3)	0 (0.0)	0 (0.0)
Rose oil (n = 5)	4 (80.0)	1 (20.0)	0 (0.0)	4 (80.0)	0 (0.0)	0 (0.0)
Geranium oil (n = 4)	3 (75.0)	4 (100.0)	3 (75.0)	1 (25.0)	0 (0.0)	0 (0.0)
Neomycin (n = 2)	0 (0.0)	1 (50.0)	2 (100.0)	1 (50.0)	1 (50.0)	0 (0.0)
Ethylenediamine (n = 2)	2 (100.0)	0 (0.0)	0 (0.0)	0 (0.0)	1 (50.0)	0 (0.0)
Sorbitan sesquioleate (n = 2)	1 (50.0)	1 (50.0)	1 (50.0)	0 (0.0)	0 (0.0)	0 (0.0)
Vanillin (n = 2)	1 (50.0)	0 (0.0)	1 (50.0)	0 (0.0)	1 (50.0)	0 (0.0)
Parthenium (n = 1)	0 (0.0)	0 (0.0)	1 (100.0)	0 (0.0)	1 (100.0)	0 (0.0)
Chlorocresol (n = 1)	0 (0.0)	0 (0.0)	1 (100.0)	0 (0.0)	1 (100.0)	0 (0.0)
Fragrance mix (n = 1)	0 (0.0)	0 (0.0)	1 (100.0)	0 (0.0)	1 (100.0)	0 (0.0)
Musk mix (n = 1)	1 (100.0)	0 (0.0)	1 (100.0)	0 (0.0)	0 (0.0)	0 (0.0)
Benzyl salicylate (n = 1)	1 (100.0)	0 (0.0)	0 (0.0)	0 (0.0)	0 (0.0)	0 (0.0)
Butylated hydroxyanisole (n = 1)	1 (100.0)	0 (0.0)	0 (0.0)	1 (100.0)	0 (0.0)	0 (0.0)
Potassium bichromate (n = 1)	0 (0.0)	0 (0.0)	1 (100.0)	0 (0.0)	1 (100.0)	0 (0.0)
Cetyl alcohol (n = 1)	0 (0.0)	1 (100.0)	0 (0.0)	1 (100.0)	0 (0.0)	0 (0.0)
Thiuram mix (n = 1)	0 (0.0)	0 (0.0)	0 (0.0)	0 (0.0)	0 (0.0)	1 (100.0)
Rubber mix (n = 1)	0 (0.0)	0 (0.0)	0 (0.0)	0 (0.0)	0 (0.0)	1 (100.0)
Total (N = 59)	33 (55.9)	24 (40.7)	22 (37.3)	21 (35.6)	8 (13.6)	2 (3.3)

## Discussion

PCD represents a distinct clinicopathological entity in which chronic, low-grade contact sensitization results predominantly in hyperpigmentation rather than overt eczematous inflammation. In the present prospective hospital-based study, patch testing proved to be a highly valuable diagnostic tool, demonstrating positivity to at least one allergen in 78.2% of patients. This high yield strongly supports the role of contact allergy in the pathogenesis of PCD and highlights the importance of systematic allergen evaluation in patients presenting with facial melanosis.

In the present study (N = 55), dermoscopy most commonly showed a brownish-grey background pigmentation (98.2%) with a brown/grey globular pigment pattern (92.7%). Follicular plugs with perifollicular white halos were also very frequent (90.9%), indicating prominent perifollicular accentuation in most cases. Linear vessels (41.8%) were seen in a moderate proportion, while mild surface scaling (25.5%) was less common, suggesting that many patients presented with a predominantly pigmentary (rather than overtly eczematous) dermoscopic picture. Superimposition of brown globules (32.7%) likely reflects increased pigment density/overlap within the affected areas.

The overall patch-test positivity rate observed in this study is comparable to that reported by Samanta et al. (2021) [7], who found positivity in 79% of patients with facial pigmented contact dermatitis using the Indian Cosmetic Series, and by Choubey et al. (2025) [8], who reported positivity in 83.3% of cases. Similar findings were also documented by Sharma et al., who observed a high rate of relevant patch-test positivity in pigmented cosmetic dermatitis [6]. In contrast, Bishnoi et al. reported a lower positivity rate (36.1%) in a broader cohort of acquired dermal macular hyperpigmentation (ADMH), suggesting that allergen detection is more frequent when PCD is studied as a distinct entity rather than being grouped with overlapping pigmentary disorders [24]. This reinforces the view that PCD represents a predominantly contact-allergic phenotype within the ADMH spectrum.

Patch-based positivity in the present study was 2.07%, which closely parallels the 3.4% reported by Samanta et al. using the Indian Cosmetic Series alone [7]. Minor variation in patch-based positivity across studies may reflect differences in the allergen panels employed, number of allergens tested, and inclusion of the Indian Standard Battery in addition to the cosmetic series. Kumar and Paulose (2014) similarly reported a patch-based positivity of 3.75% in cosmetic allergic contact dermatitis, further validating the magnitude of findings in the present study [22]. Collectively, these observations underscore patch testing as a sensitive and indispensable tool in the evaluation of pigmentary contact dermatitis.

With regard to allergen distribution, PPD emerged as the most common allergen in the present study, accounting for 27.1% of all positive reactions. This was followed by cetrimide (15.3%), lavender absolute (11.9%), rose oil (8.5%), and geranium oil (6.8%). The predominance of PPD is in strong agreement with findings by Samanta et al., Choubey et al., and Sharma et al., all of whom identified PPD as the leading allergen in Indian cohorts of PCD [6-8]. Sharma et al. additionally highlighted the rising relevance of hair dye-related sensitization in pigmentary dermatoses, reflecting increasing cosmetic use and availability of PPD-containing products [6].

Earlier Indian studies such as those by Nath and Thappa (2007) reported thimerosal and gallate mix as dominant allergens, with PPD being less frequent [17]. This temporal shift suggests changing cosmetic formulations, increased popularity of hair dyes and fairness products, and evolving exposure patterns over time. The frequent detection of cetrimide/cetrimonium-type compounds in the present study aligns with observations by Sharma et al., who reported cetrimonium bromide as a common sensitizer in pigmented cosmetic dermatitis, reflecting the widespread use of such compounds as preservatives and antiseptics in topical formulations [6].

Analysis of exposure sources among patch-test-positive cases further strengthens the etiological relevance of identified allergens. Hair dye exposure was the most frequent source (55.9%), followed by fairness creams (40.7%), cosmetic products (37.3%), and mehendi (35.6%). This distribution mirrors the exposure patterns reported by Samanta et al. and Choubey et al., both of whom emphasized hair dye as the single most important exposure in facial PCD [7,8]. Subburaj also highlighted hair dyes, fragrances, and fairness creams as major contributors in Indian PCD, reinforcing the cultural and cosmetic context of sensitization [25].

Importantly, PPD showed strong clustering with hair dye and mehendi exposure, as well as significant associations with fairness creams and cosmetic products, confirming its central role in the pathogenesis of PCD. Similar exposure-allergen relationships were described by Samanta et al., who noted that most PPD-positive patients had a history of hair dye use, and by Choubey et al., who linked fragrance allergens predominantly with fairness creams and perfumed cosmetics [7,8]. Essential oils such as lavender and rose oil, which were frequently detected in the present study, have also been recognized by Bhat et al. and Subburaj et al. as emerging cosmetic sensitizers contributing to pigmentary contact dermatitis [10,25].

The analysis of patch-test reaction strength revealed that most reactions were weakly positive, and no statistically significant association was found between allergen type and reaction strength (χ² p = 0.494). This pattern is characteristic of PCD and has been emphasized by Subburaj et al., who described PCD as a low-grade, pigment-dominant hypersensitivity disorder where mild (+) reactions are common yet clinically relevant [25].

Both Samanta et al. and Choubey et al. similarly reported predominance of weak-positive reactions using ICDRG grading, reinforcing that strong eczematous responses are not required to produce clinically significant pigmentation [7,8].

In summary, the present study confirms that PPD-containing hair dyes remain the most important etiological factor in pigmented contact dermatitis, with additional contributions from preservatives and fragrance constituents present in fairness creams, cosmetics, and traditional products such as mehendi. The high rate of patch-test positivity, predominance of weak-positive reactions, and clear exposure-allergen correlations underscore the need for meticulous history taking and systematic patch testing in all patients with facial melanosis suspected to be PCD. Early identification and avoidance of relevant allergens are crucial, as pigmentation often persists for prolonged periods despite cessation of inflammation, making prevention and patient education central to long-term management.

Limitations of our study include the lack of blinding and the single-centre design. Exposure data were patient-reported; cumulative dose, frequency and latency could not be reliably quantified, as patients typically use multiple cosmetics interchangeably over years and traditional preparations such as sindoor and mehendi carry no standardised dosing measures. Independent ingredient verification was not feasible, as Indian cosmetic regulations do not mandate full labelling on traditional and locally manufactured products. Repeat open application testing was not undertaken; pigmentary endpoints in PCD evolve over months, and protocolised re-exposure is undesirable in actively pigmenting disease, and the close topographic correspondence between pigmentation and reported product use was therefore used as a surrogate for relevance. Short follow-up precluded assessment of post-avoidance pigmentary change.

## Conclusions

The findings of this study underscore the need for a high index of clinical suspicion in patients presenting with facial hyperpigmentation, detailed exposure history, and routine use of standardized patch testing to identify relevant allergens. Early recognition and strict avoidance of offending agents are crucial, as pigmentation may persist for prolonged periods even after allergen withdrawal. Furthermore, the study underscores that weak positive patch test reactions should not be disregarded, as they can provide valuable diagnostic evidence for pigmented contact dermatitis despite the absence of strong vesicular responses. Documenting locally relevant allergen patterns can aid in patient counseling, guide preventive strategies, and contribute to improved formulation and regulation of cosmetic products, ultimately helping to reduce the burden of pigmented contact dermatitis in the community.
